# A Review of *Streptococcus pyogenes*: Public Health Risk Factors, Prevention and Control

**DOI:** 10.3390/pathogens10020248

**Published:** 2021-02-22

**Authors:** Nelly Janira Avire, Harriet Whiley, Kirstin Ross

**Affiliations:** Environmental Health, College of Science and Engineering, Flinders University, Adelaide 5001, Australia; janiravire@gmail.com (N.J.A.); harriet.whiley@flinders.edu.au (H.W.)

**Keywords:** group A strep, group A streptococcus, Strep A, GAS, streptococcus pyogenes, risk assessment, infection control, management, policy

## Abstract

*Streptococcus pyogenes,* (colloquially named “group A streptococcus” (GAS)), is a pathogen of public health significance, infecting 18.1 million people worldwide and resulting in 500,000 deaths each year. This review identified published articles on the risk factors and public health prevention and control strategies for mitigating GAS diseases. The pathogen causing GAS diseases is commonly transmitted via respiratory droplets, touching skin sores caused by GAS or through contact with contaminated material or equipment. Foodborne transmission is also possible, although there is need for further research to quantify this route of infection. It was found that GAS diseases are highly prevalent in developing countries, and among indigenous populations and low socioeconomic areas in developed countries. Children, the immunocompromised and the elderly are at the greatest risk of *S. pyogenes* infections and the associated sequelae, with transmission rates being higher in schools, kindergartens, hospitals and residential care homes. This was attributed to overcrowding and the higher level of social contact in these settings. Prevention and control measures should target the improvement of living conditions, and personal and hand hygiene. Adherence to infection prevention and control practices should be emphasized in high-risk settings. Resource distribution by governments, especially in developed countries, should also be considered.

## 1. Introduction

*Streptococcus pyogenes*, (colloquially named “group A streptococcus” (GAS)), is a Gram-positive bacterial pathogen that can cause both non-invasive and invasive disease (iGAS), as well as nonsuppurative sequelae. This includes pharyngitis, scarlet fever, impetigo, cellulitis, type II necrotizing fasciitis, streptococcal toxic shock syndrome, acute rheumatic fever and post-streptococcal glomerulonephritis [1]. Approximately 18.1 million people currently suffer from a serious GAS disease, with 1.78 million new cases and 500,000 deaths occurring each year [2].

During the 20th century there was a decrease in the incidence of GAS diseases in developed countries, largely as a result of improved living conditions [3]. However, genetic changes in circulating GAS strains and/or changes in host susceptibility to infection can lead to dramatic increases in the rates of specific diseases [4,5,6]. No situations exemplify this more than the global upsurge of invasive GAS disease that originated in the 1980s, and the regional increases in scarlet fever in Northeast Asia and the United Kingdom [7,8]. In each case, increased disease rates have been associated with the emergence of new GAS strains with increased disease-causing capability. Epidemiological studies also show the re-emergence of these diseases in developed countries. Studies from the United States have indicated that iGAS infection rates from 2005 to 2012 remained steady, with 3.8 cases per 100,000 persons, and resulted in 1116 deaths per year [7]. In 2015, the United States reported >15,000 cases of iGAS disease and 1600 deaths [9]. GAS disease cases have also been reported to increase over time in Canada. The rate for GAS infections in Canada in 2015 was 5.24, up from 2.4 cases per 100,000 persons in 2003 [10]. In the United Kingdom, the incidence rate of iGAS disease has been reported to be 2.9 cases per 100,000 persons per year [11]. Typically, GAS infections and the associated sequelae are more prevalent in areas of socioeconomic disadvantage [12,13,14]. This includes developing countries, and the socioeconomically disadvantaged populations within developed countries [10,15,16]. 

There is an urgent need to improve public health strategies to prevent the transmission of GAS diseases, particularly within vulnerable populations. The aim of this review was to identify all the population risk factors and potential public health prevention and control strategies. Identifying the factors increasing the risk of transmission will inform public health policy and reduce the spread of GAS diseases. 

## 2. Results

An overview of the public health risk factors, and the prevention and control strategies for GAS diseases found in the reviewed articles, are presented in Table 1.

### 2.1. Areas of Prevalence

Group A streptococcus (GAS) diseases were reported to be common in developing countries [17,18,19] and among the indigenous populations in developed countries [10,15,20,21,22,23,24,25,26,27,28,29], and reported to be endemic in low socioeconomic communities [9,20,30]. GAS diseases are also present in high socioeconomic communities, in conditions where people are in close proximity to one another for extended periods of time (for example, schools and care facilities) [8,36,37,38,42,43,44,45,46,47,48].

### 2.2. Mode of Transmission

The mode of transmission for GAS infection was reported to be primarily through respiratory droplets and direct contact with infected people and surfaces [16,31,32,33,34]. However, contaminated food was identified as another possible mode of transmission [35].

### 2.3. At-Risk Groups

The population groups at most risk of being infected with GAS diseases included people with underlying medical conditions [10,17,27,59,60,61,62], children (0 to 15 years) [8,11,36,51,52,53,54,55,56] and the elderly [10,43,57,58]. Additionally, pregnant and postpartum women were also identified as groups at risk for GAS diseases [39,41,63,64,65,66,67,68,69]. However, in the wider community, when gender was considered, boys and men were reported to be more at risk of being infected with GAS compared to girls and women [22,23,36,52,57,70].

### 2.4. Common Environments of Exposure

The common environments for GAS infections included schools, nurseries and kindergartens [16,36,37], hospitals [33,34,38,39,40,41], homeless shelters [22,50], care homes [38,42,43,44,45,46,47,48] and military training facilities [49].

### 2.5. Risk Factors

Inadequate housing was identified as a major risk factor for GAS diseases. This includes homelessness [10,28,62,70,71,72,73], household overcrowding [20,51], limited household resources (including the sharing of personal items) [15,71], and poor housing conditions, such as dampness, poor ventilation and house temperature [18,20]. Low socioeconomic status [9,20,30], environmental tobacco smoke exposure [53,120] and exposure to biting insects or skin injuries or diseases were also identified as risk factors [15,60,71,112,121]. Several studies noted seasonal variations in the prevalence of GAS diseases [8,52,53,57].

Other factors identified as promoting the spread of GAS infections, especially in hospitals and care homes, included poor infection control practices resulting in cross-infection by healthcare workers, other patients or residents, and the cross-contamination of hospital equipment and devices [31,32,39,88,89,90,91,92,93]. Poor personal and hand hygiene [15,42] and exposure to asymptomatic persons [77] were also highlighted as risk factors for GAS infections. These risk factors often exasperated the high likelihood of transmission occurring between household contacts [11,37,64,77]. Other individual risk factors include change of host immunity due to conditions such as pregnancy [39,41,63,64,65,66,67,68,69], underlying medical conditions [10,17,27,39,41,59,60,61,62,63,64,65,66,67,68,69], malnutrition [17], and illicit drug use or alcohol abuse [62,72,73].

### 2.6. Prevention and Control Strategies

Several studies have suggested that early diagnosis and treatment is an effective way to prevent and control iGAS infections and complications from mild infections [15,19,69,79,80,81,82,83,84,85,86,87]. In hospitals and care facilities, effective infection control procedures were identified as being crucial for the prevention of GAS infections [31,32,39,88,89,90,91,92,93]. This could also include the screening of asymptomatic cases, including healthcare workers, and the post-exposure prophylaxis of vulnerable groups [32]. The need for improved surveillance and epidemiological investigation was also emphasized by several articles [111,112,113,114,115,116]. This would be supported by improved detection methods [105,106] and health education at the hospital and community levels, including the capacity building of healthcare workers and education on the proper diagnosis and management of GAS diseases [15,29,45,85,100,101,102,103,104]. 

Other prevention and control measures include improving the quality of housing [15,20], improved hand hygiene practices [15,42] and the reduction of overcrowding [15]. Other proposed methods to reduce GAS transmission that are not currently used include vaccination [89,102,107,108,109,110] and screening for GAS during pregnancy [41,63,67,97,119]. 

## 3. Discussion

### 3.1. Areas of Prevalence

Despite being in existence for hundreds of years, GAS still creates a substantial burden of disease and death on a global scale, mainly in children and young adults in less developed countries [122]. In low- and middle-income countries, and in disadvantaged populations in high-income countries such as the United States and Australia, GAS diseases remain endemic [9,14]. GAS diseases also remain relatively important in more developed countries. According to this review, the broad diversity of GAS genotypes has led to the persistence of these infections globally [12]. Strain variations also exist for GAS between developed and developing countries. Low-income settings report high GAS strain diversity compared with high-income settings. The reason as to why this is so is not clear [123]. However, Tartof et al. [18] argue that local factors, such as crowding, may enhance the frequency of GAS transmission and horizontal gene transfers that contribute to the increased strain diversity in such settings. A study carried out in South Africa showed that there were similarities in the GAS strains when they were compared with the ones in Tunisia and Kenya, but they were different when compared with those of developed countries [107]. Similarities in strain diversity were also reported in settings with similar living conditions, which include indigenous populations in developed countries [9,56]. This review also found that socially disadvantaged communities are heavily burdened by these diseases due to their low socioeconomic status, characterized by poor housing conditions and inability to afford medical care, among other factors [15,20,21,26,118,124]. According to a study performed in the United Kingdom, interviews conducted with the patients, teachers and parents of children affected with rheumatic fever revealed that low socioeconomic status was common among most of the respondents [51]. In Australia, records indicate that GAS infections and their sequalae among Indigenous Australians continue to persist at equal or higher levels when compared with cases in developing countries [14]. Tropical regions of northern Queensland and the Northern Territory, the only states that report GAS infections as notifiable diseases, have the highest reported rates, with the incidence in indigenous populations ranging from 23.9 to 82.5 cases per 100,000 persons, and in non-indigenous populations from 4.7 to 10.3 cases per 100,000 persons. This has been linked to the higher proportion of indigenous people in northern Australia who experience high levels of socioeconomic disadvantage and household overcrowding [27]. In Alaska in the United States, a region where indigenous Americans live, the incidence rate of GAS infections in 2015 was 12.3 cases per 100,000 persons, a rate that was more than twice that of the rest of the United States [9].

A limitation in identifying the areas of highest prevalence is the fact that GAS diseases are not always notifiable. A few countries, such as England, Wales, Japan, Canada, Norway, China and the United States, report specific GAS diseases, but not all clinical manifestations. For example, in Japan and the United States, only streptococcal TSS is notifiable [6,125], in Canada and Norway all iGAS diseases are notifiable [10,57], and in China only scarlet fever is notifiable [96]. In other countries, the disease is only notifiable in specific regions. For example, in Australia, GAS diseases are notifiable in the Northern Territory and Queensland only [15]. Another limitation with this review is that only articles published from 2010 to 2020 were included. This may have introduced a bias towards publications from Asia and Australasia, since an increasing number of publications on this subject have originated from these regions during the last few years.

### 3.2. Transmission

Understanding the transmission routes is essential for identifying appropriate control interventions. The individual transmission routes for each GAS disease are shown in Table 2. Humans are the only natural reservoir for GAS, and it is commonly found in the carrier state on the anus, vagina, pharynx and skin of human hosts without causing disease [126]. 

Transmission of GAS primarily occurs through respiratory droplets, or skin contact with broken skin that has secretions from infected sores on it [31,127,128]. The environment is also a potential reservoir, and facilitates transmission through contaminated equipment, surfaces, dust and fomites [16,31,32,33,34]; however, very few current studies have explored this area, hence it is an area for further research. The foodborne transmission of GAS infections is also possible [35]. GAS has been shown to survive in ice cream (18 days), raw and pasteurized milk at 15–37 °C (96 h), room temperature butter (48 h) and neutralized butter (12–17 days) [129]. Additionally, GAS has been found to last several days in salads at room temperature [130]. An investigation of a GAS outbreak in China in 2014 among a film crew demonstrated that foodborne outbreaks due to GAS infections exist, although they are very rare. However, these outbreaks are always difficult to recognize at early stages, and thus are usually ignored by healthcare workers [131]. From the literature reviewed, very few studies have been done with regards to foodborne GAS transmission, and therefore more research needs to be done on the same. 

### 3.3. Common Areas of Infection

GAS infections are commonly spread in schools, nurseries and kindergartens, hospitals, care homes, military camps and homeless shelters [8,22,33,34,36,37,38,39,40,41,42,43,44,45,46,47,48,49,50]. A review of epidemiological data for scarlet fever for the period 2005–2015 conducted by Zhang et al. [8] in Hong Kong showed that the infections were higher during the months when schools were open. Additionally, more cases were reported among children who attended nurseries and kindergartens. This was attributed to low immunity in this population group and high populations in these settings [8]. 

Cummins et al.’s [42] review of 20 outbreaks in care homes showed high infection rates existed as a result of cross-infection from infected care home residents to the healthy residents, as well as infections from home care staff to residents. However, cross-infection from care staff was slightly lower compared with cross-infection among residents. This is supported by Dooling et al. [47] and Deutscher et al. [65]. The incidence of GAS diseases in residents of long-term care facilities is also higher (3-8-fold) than among community residents of the same age [43].

This review identified hospitals as high-risk areas for infections [33,34,38,39,40,41]. This was attributed to poor surgical procedures, contaminated medical instruments or hospital environments, and cross-infection from other patients and healthcare workers [33,38,39,44,74,75]. 

According to Engelthaler et al. [22], evidence from a study conducted on clients from homeless shelters and jails in the United States showed that conditions in these settings favored the transmission of GAS infections. A comparable study conducted in Canada also reported similar findings [62]. Hammond-Collins et al. [49] also conducted a study on GAS-infected cases between August 2016 to January 2018 in Belgium. The results showed incidence rates of 2333/100,000 persons in homeless groups and 25/100,000 persons in non-homeless groups, showing a higher incidence (100 times higher) for homeless persons compared to non-homeless persons. An outbreak investigation in homeless shelters done in Canada in 2019 supported the same findings [50].

### 3.4. At Risk Groups

GAS affects anyone in any population [132,133]. However, GAS diseases are more common in children and the elderly [8,10,11,36,43,51,52,53,54,55,56,57,58], as demonstrated in this review. School-aged children were consistently identified as high-risk for GAS diseases. A population-based case control study undertaken in New Zealand between 2010 and 2014 showed that 79.1% of new cases were reported in children 5–17 years old, and cases were rare in children 4 years old and below [121]. This was also reported in studies from the United Kingdom in 2013, where most cases were children 5 to 14 years old [51]. Additionally, a review of scarlet fever cases for the period 2005–2015 in Hong Kong revealed a high incidence among children 5–15 years old, although those at most risk were children 3–5 years of age. This age group includes children just entering kindergarten [36]. Even though any person of any age group can be an asymptomatic case of a GAS disease, most asymptomatic cases are seen in children. An asymptomatic person can act as a reservoir for GAS, and therefore pose a greater risk of transmission [80]. 

In addition to children, the elderly population are high-risk for GAS diseases [10,43,57,58]. A study of the review of outbreaks of GAS diseases in Europe conducted by Cummins et al. [42] established that 20 out of 31 outbreaks that occurred between 1992 and 2008 were related to residential care homes. Chalker et al.’s [38] study also showed that many cases of GAS infections were common in the elderly population, especially those over 70 years. Mearkle et al. [64] demonstrated an increased risk of GAS transmission within a household when one family member is positive. This risk was greatest in couples aged 75 years and older, and in mother–neonate pairs.

People with underlying medical conditions are also more vulnerable to GAS infections, as seen in the reviewed articles [10,17,27,59,60,61,62]. This review also showed that previous skin conditions and recent wounds were also some of the medical conditions that favored the transmission of GAS infections [50,71]. Pre-existing medical conditions and co-infections such as influenza, malnutrition, diabetes mellitus, HIV and malaria also expose people to GAS infections, due to a reduced immunity for fighting infections [10,17,27,59,60,61,62,78], as seen in the reviewed articles. Sosa [127] argues that pre-existing medical conditions in pregnancy can cause GAS infections to progress to toxic shock syndrome or necrotizing fasciitis, severe types of GAS diseases. This review also reported changes in host immunity, especially during pregnancy, as a risk factor for GAS infections [39,41,63,64,65,66,67,68,69]. The results of a study carried out by Rottenstreich et al. [67] reported that pregnant women were 20 times more at risk of GAS infections than non-pregnant women. This has been attributed to changes in host immunity due to pregnancy or postpartum status [103,119]. Studies carried out on pregnant women also reveal that GAS infections can cause still births and neonatal deaths [65]. Additionally, caesarean sections undertaken with contaminated medical instruments expose pregnant women to GAS infections [127]. Lactation also reduces the availability of protective vaginal flora such as lactobacillus, and thus increases the chances of the growth of other microorganisms such as GAS [134]. Increased incidence has also been reported in infants mainly through exposure to asymptomatic persons in the households, or mother to neonate cross-infection [64].

In terms of gender, the review showed that higher incidence rates of GAS infections are reported in men compared with women [22,23,36,52,57,70]. According to a study carried out in China, the incidence of scarlet fever cases from 2004 to 2016 was 1.54 times greater among boys compared with girls and women [52]. Lee, Cowling and Lau [36] attribute this high risk to more physical interactions and poorer personal hygiene among boys. In the United Kingdom, however, there is an even distribution of cases across all genders [43]. Very little is known about why the incidence is higher in men than in women.

### 3.5. Risk Factors for GAS Infections

The transmission of GAS is determined by a number of factors. GAS is chiefly a disease of poverty [3]. Housing conditions characterized by overcrowding, dampness, poor ventilation and/or lack of temperature control encourage the transmission of these infections [18,20,51]. Overcrowding, especially in households, in military camps and in other institutions such as care homes, is a significant environmental factor for the spread of GAS infections [18,51,64], as seen in the reviewed articles. In overcrowded conditions, coughing or sneezing from one infected person in the household or institution can easily infect others [135]. Since these bacteria are believed to survive on dry surfaces and materials for up to 6.5 months, there is an increased likelihood of their transmission in overcrowded settings [136]. 

Contamination in hospitals and nursing facilities is also a risk factor that the review highlighted, and this needs to be addressed if infections are to be prevented or controlled. This contamination includes shared hospital equipment, surroundings such as curtains, furniture, walls and floors and devices, or implants [33,74,75]. Contamination from healthcare workers due to poor infection control practices can also occur [38,39,44,45].

Substandard infection control practices, including errors in equipment sterilization, lack of cleaning and disinfection of shared hospital equipment, lack of proper use of personal protective equipment, poor waste management and disposal and poor wound care practices, are also major contributors to the transmission of GAS infections [32,48,74,88]. For example, in a study performed by Mahida et al. [33] in an ear, nose and throat ward in a hospital in the United Kingdom, ward curtains sampled and tested for GAS during an outbreak in a hospital showed that 10 out of 34 curtains tested positive for GAS.

Cross-infection by health workers colonized with GAS to patients was reported to occur in hospitals [77]. Exposure to asymptomatic persons or cases of GAS infections can also occur at the household level. Such exposure is high in overcrowded households, as discussed earlier. In addition, limited household resources, such as those for washing and laundry, contribute to an increase in bacterial load on the skin of household members, or objects in the house, resulting in increased transmission. Moreover, sharing bedding and personal items like towels is also a predisposing factor for the transmission of GAS infections [121]. High numbers of social contacts, a key environmental factor, which is common in schools, hospitals and other enclosed social places, increase the chances of the transmission of GAS infections [49,137]

Personal hygiene and hand hygiene are key to the control and prevention of communicable diseases like common cold, diphtheria, rubella and GAS infections [33,48]. Poor personal and hand hygiene have been proven to be risk factors for GAS infections in all age groups [15,42]. However, school-going children, especially boys, have been reported to be highly susceptible. This has been attributed to lower hygiene standards among the boys [36]. Poor hygiene practices like infrequent tooth brushing among people living in homeless shelters also contributes to an increased risk of GAS spread among these population groups [71]. Broken skin was also identified as a risk factor for GAS transmission. Healthy skin provides a barrier of protection against infections, and when broken, it provides a good growth environment for GAS, hence the high chances of infection [23,117]. GAS biofilms can also form on human tissues, especially in necrotizing soft tissue infections [138]. 

Exposures to tobacco smoke and other air pollutants are risk factors for GAS infections [53,111] that compromise the immune system, thus increasing the risk of infection. 

Knowledge gaps on the proper diagnosis and management of GAS infections among health workers still exist. For example, a study carried out in Italy in 2017–2018 on pediatricians’ knowledge of the diagnosis and management of GAS infections showed that only 8% of 154 pediatricians correctly answered all of the questions on GAS diagnosis and management. Understanding diagnosis and management is particularly important in reducing the incidence of GAS sequelae [101].

Seasonal variation was also reported as a factor that influenced the transmission of GAS infections. Higher case numbers have been reported during winter and the early spring months [8,53,57].

Alcohol abuse and intravenous drug use are also risk factors for GAS infections [62,72,73]. However, not all GAS disease cases found in populations that inject drugs or abuse alcohol are directly linked to these factors. An outbreak investigation carried out in England among cases of GAS infection who were injecting-drug users showed that the transmission of these infections was not associated with the alcohol or drug use [73]; however, very few studies have explored this factor, and this therefore presents an area for future research.

### 3.6. Prevention and Control Measures/Strategies

Strategies aiming to prevent or treat GAS infections should be feasible, accessible and affordable, especially in low-resource settings [3]. The prevention and control of GAS infections has been approached from the public and environmental health and clinical perspectives; however, most of the intervention programs available focus more on clinical intervention, and there are limited data on possible infection prevention strategies in the community [114]. The available public health strategies focus on minimizing transmission and the protection of the people most vulnerable to GAS infections in all areas with increased potential for infection. Primary preventive strategies are also necessary, since they prevent irreversible health conditions that may arise from complications due to GAS infections [118]. These strategies include epidemiological investigations and improved surveillance systems [94,95,96,97,98,99,111,112,113,114,115,116], improved quality of housing [18,20], good hand hygiene, which includes regular proper hand washing with soap and water, or use of alcohol hand rub [15,42], and avoiding overcrowding [15]. Improved personal hygiene is also key in controlling transmission, especially in boys, who tend to be more at-risk than girls [36]. Limited or no sharing of personal items like towels and even beddings should be encouraged to reduce the spread of GAS infections [15]. The sharing of items that could be contaminated with saliva, such as water bottles, drinking glasses, utensils, etc., should also be avoided [71].

Environmental sanitation, including the cleaning and sanitation of surfaces and common touch areas, should be maintained in all areas considered risky transmission zones [34,139]. GAS diseases have been reported to be susceptible to moist heat of 121 °C for at least 15 min, and dry heat of 170 °C for at least 1 h. In addition, the bacteria are also susceptible to 1% sodium hypochlorite, 4% formaldehyde, 2% glutaraldehyde, 70% ethanol, 70% propanol, 2% peracetic acid, 3–6% hydrogen peroxide and 16% iodine [140]. The constant disinfection and cleaning of shared equipment, especially in hospitals, should be encouraged [47,74]. This review also highlights decontamination and the thorough cleaning of curtains and communal facilities, such as bathrooms and toilets, as key measures in the prevention of infections such as GAS infections [39,141]. Curtains in high-risk areas such as hospital settings should also be changed frequently. This should be done once a month in high-risk areas and in low-risk areas twice a year. Hospitals should also consider using disposable curtains or plastic screens instead of washable curtains [33].

Appropriate infection control practice was also identified as critical to preventing GAS infections in hospital and healthcare settings. This includes the proper use of personal protective equipment, aseptic management of wounds and proper disposal of medical waste [31,32,39,88,89,90,91,92,93]. Patients or residents infected with GAS in hospitals or care homes should also be isolated as a prevention measure to stop further GAS transmission [93,104]. The support of antisepsis measures during delivery and neonatal cord care was also identified as important to the prevention strategy to reduce GAS transmission among birthing women and neonates [17]. Medical practitioners are also required to adhere to the diagnosis and treatment guidelines to effectively control GAS infections [35,101,124]. This review also advocates for the proper management of co-infections, such as influenza [78], and patients who have undergone surgical interventions, such as tonsillectomy and other similar operations [40,142], so as to reduce the risk of the development of GAS infections.

The screening of health workers, asymptomatic cases including social contacts and family members of infected persons, and post-exposure prophylaxis for vulnerable groups is encouraged [11,32,37,64,77]. Successful screening should include both the throat and perianal sites [75]. However, the lack of official guidelines concerning the prevention of secondary disease using contact prophylaxis remains a challenge in many countries [111]. In addition, chemoprophylaxis can sometimes be ineffective, especially in controlling outbreaks due to the introduction of new strains as a result of the mutating nature of GAS [48]. 

Health education for healthcare providers, patients and communities is key for the prevention of GAS infections [15,45,85,100,101,102,103,104]. This should include messages that encourage people to cover coughs or sneezes with a tissue or a forearm, which is effective in the prevention of most infections transmitted through respiratory droplets [49,143]. Messages on proper health-seeking behavior should also be emphasized, since this also helps to reduce disease spread through treatment success [19,49,100]. The capacity building of health workers as regards GAS infections and their control will also help to provide targeted advice to clients, and thus break the transmission chain [3,14,26,85]. This will reduce existing knowledge gaps in GAS infections prevention and management. After training is completed, facilities should try to avoid high staff turnover, which is likely to contribute to knowledge gaps in the control and management of these infections [47].

Clinical interventions, such as early accurate diagnosis and treatment as an effective preventive measure for GAS infections, were highly advocated for [15,19,69,79,80,81,82,83,84,85,86,87]. Early treatment with antibiotics is essential for reducing the transmission window. It also prevents the development of complications, including associated sequelae. For example, with antibiotics, GAS is typically communicable for 24–48 h; however, without antibiotics, communicability can last for 10–21 days, and even longer in complicated cases [144]. This is illustrated by an outbreak of GAS pharyngitis in a Canadian military camp between December 2016 and April 2017. The outbreak investigation found that reluctance to seek medical care and low compliance with antibiotics were reported factors that hindered treatment success and led to the increased spread of the GAS disease [49]. Patients are therefore advised to adhere to the treatment advice to avoid treatment failure, and therefore reduce disease spread. In addition, the early and improved treatment of skin infections and burns has been encouraged as a prevention measure [14,28,71,145]. Safe injection practices should also be encouraged among people who inject drugs [146] to reduce GAS transmission rates in these populations.

Improved detection methods and outbreak investigation tools, such as whole genome sequencing, can enable the early detection of GAS infections and the identification of outbreaks. This can facilitate the rapid administration of treatment, and reduce the transmission rate [105,106]. Governments should also ensure the availability of affordable and accessible healthcare services to all citizens to prevent and control all infections. Equitable resource distribution is key in reducing social disadvantage, which highly influences GAS transmission [3,9,20,30].

Currently, there is no licensed vaccine for the control of GAS infections; however, the development of a vaccine is underway [2]. This process has been hindered by factors like the availability of various unique GAS serotypes, antigenic variations within the same serotype, safety concerns, and a lack of consensus on clinical endpoints for the establishment of proof of concept [108,110,147,148]. Studies in this review, however, indicate that vaccination could help reduce these diseases, and therefore highly recommend its development and use [89,102,107,108,109,110]. Rivera-Hernandez et al. [109] suggest that vaccination can reduce antibiotic use and therefore reduce antibiotic resistance. Reducing antibiotic resistance will further reduce the transmission of GAS infections through increased treatment success, as discussed earlier. Studies have also proposed screening for invasive GAS during pregnancy so as to prevent transmission to pregnant women who are a high-risk group for GAS infections [41,63,67,97,119].

GAS is an important global pathogen with diverse clinical manifestations and limited epidemiological data [3,122]. Currently, a lack of mandatory patient notification in most countries limits the ability of public health programs to effectively target, prevent and control GAS diseases [111,112,113,114,115,116]. Most studies and countries focus more on the clinical management of the diseases than on prevention at the community level. Since GAS diseases are not notifiable worldwide, conducting informed public health and research initiatives aimed at reducing the impact of GAS diseases remains a challenge. Efforts to improve reporting systems, and scaling up public and environmental health interventions at the community level coupled with the effective treatment of cases, will help reduce the transmission rate [46,94,111,113,122]. Future strategies must use a multi-disciplinary approach to the prevention and control of GAS diseases in both the community and clinical settings.

## 4. Materials and Methods 

The databases Scopus and Web of Science were searched for articles written in English over the last ten years with the following keywords: group a strep; group A streptococcus; Strep A; GAS; Streptococcus pyogenes; S. pyogenes; Group A streptococci AND control; prevention; public health; infection control; intervention*; management; risk factor*; risk*. Articles were screened by reading titles and abstracts and initially excluded if they did not refer specifically to group A streptococcus or *Streptococcus pyogenes*, or if they were review articles or not written in English. Further screening was done by reading the text in full to exclude articles that did not describe either at-risk groups for the spread of these infections, risk factors for the spread of these infections or the prevention and control strategies of these infections. Any studies that focused on the laboratory isolation of different GAS genotypes only and the treatment regimen for GAS infections were also excluded. Since the aim of this study was to provide overall knowledge on GAS infections, risk factors for their spread, as well as prevention and control strategies, all studies that met the inclusion criteria were included regardless of any perceived faults in the study design. 

## 5. Conclusions

Globally, GAS infections and their sequalae represent a significant public health issue. However, GAS diseases are most prevalent in developing countries, and among indigenous populations and low socioeconomic areas in developed countries. Public health policy should focus on preventing the transmission of GAS. This primarily occurs through respiratory droplets, and direct contact with skin sores caused by GAS or contaminated material or equipment. Future research is needed to explore the significance of foodborne transmission and other environmental sources. The incidence of GAS diseases is particularly high in school-aged children, and this should be the focus of intervention strategies, as GAS diseases can affect education and consequently their societal development, creating a cycle for the disease and disadvantage. To break this cycle, public health community-based preventive measures need to be strengthened. Making GAS diseases notifiable at the national level would help to inform public health and research initiatives to reduce the impact of these diseases. Governments also need to consider greater equity in the distribution of resources, in order to raise living standards and thus reduce the burden of communicable diseases such as GAS infections. Moreover, action should be taken to ensure everyone has access to quality healthcare, as early diagnosis and treatment can reduce the transmission window for GAS and prevent disease complications.

## Figures and Tables

**Table 1:** Synthesis of the key findings relating to the risk factors for GAS disease(s) and the public health prevention and control strategies.

Key Findings or Conclusion	Risk or Strategy	References
Common areas of prevalence	Developing countries	[17,18,19]
	Indigenous communities in developed countries	[10,15,20,21,22,23,24,25,26,27,28,29]
	Low socioeconomic communities	[9,20,30]
Mode of transmission	Respiratory droplets and contact with infected persons or surfaces	[16,31,32,33,34]
	Contaminated food	[35]
Common areas of exposure to GAS	Schools, nurseries and kindergartens	[8,36,37]
	Hospitals	[33,34,38,39,40,41]
	Care homes	[38,42,43,44,45,46,47,48]
	Military training facilities	[49]
	Homeless shelters	[22,50]
At-risk groups	Children (infants to 15 years old)	[8,11,36,51,52,53,54,55,56]
	Elderly	[10,43,57,58]
	People with underlying medical conditions	[10,17,27,59,60,61,62]
	Pregnant women, women with postpartum status and neonates	[39,41,63,64,65,66,67,68,69]
	Gender—Boys and men	[22,23,36,52,57,70]
Risk factors for GAS infections	Household crowding	[20,51]
	Poor housing conditions—dampness, temperature, poor ventilation	[18,20]
	Homelessness	[10,28,62,70,71,72,73]
	Hospitalization or hospital equipment	[33,74,75]
	Cross-infection by healthcare workers	[38,39,44,76]
	Exposure to asymptomatic persons	[77]
	Household contact	[11,37,64,77]
	Limited household resources—including those of washing, teeth cleaning and laundry	[15,71]
	Environmental tobacco smoke exposure and other air pollutants	[20,53]
	Exposure to biting insects and skin injuries	[50,71]
	Seasonal variation	[8,52,53,57]
	Co-infection with other infections (e.g., influenza)	[78]
	Illicit drug use or alcohol abuse	[62,72,73]
Prevention and control measures/strategies	Early/improved diagnosis and treatment	[15,19,69,79,80,81,82,83,84,85,86,87]
	Infection control in hospitals/aged care settings	[31,32,39,42,88,89,90,91,92,93]
	Epidemiological investigations/improved surveillance systems	[94,95,96,97,98,99]
	Improved quality of housing	[15,20]
	Health education for healthcare providers and patients/community	[15,45,85,100,101,102,103,104]
	Screening of healthcare workers, asymptomatic cases and post-exposure prophylaxis for vulnerable groups	[32]
	Hand hygiene	[15,42]
	Avoid overcrowding	[15]
	Reduced malnutrition and HIV infection	[17]
	Improved GAS detection methods	[105,106]
	Capacity building of healthcare workers	[29]
Suggested future prevention and control strategies	Vaccination	[89,102,107,108,109,110]
	Improved surveillance/mandatory case notification	[111,112,113,114,115,116]
	Education and engagement	[117]
	Primary prevention strategies	[118]
	Screening for GAS during pregnancy/postpartum	[41,63,67,97,119]

**Table 2:** Transmission routes for diseases caused by *Streptococcus pyogenes.*

Diseases Caused by Streptococcus Pyogenes	Transmission Route
**Direct infection**	
Pharyngitis (strep throat)	Direct person-to-person transmission, typically through saliva or nasal secretions
Cellulitis	Direct person-to-person transmission typically through contact with skin lesions or exposure to respiratory droplets
Impetigo	Direct person-to-person transmission
**Toxin-mediated disease**	
Scarlet fever	Direct person-to-person transmission, typically through saliva or nasal secretions
Necrotizing fasciitis	In necrotizing fasciitis, the initial entry of group A strep into the body can occur by several routes. This includes non-penetrating trauma (e.g., bruises, muscle strain), causing seeding from transient bacteremia, and penetrating trauma, enabling the bacteria to pass directly to the site of infection from the environment.
Streptococcal toxic shock syndrome (STSS)	Through a compromised barrier (such as a skin injury) or through mucus membranes. The bacteria then spread to deep tissues and eventually to the bloodstream.
**Immune-mediated disease**	
Acute rheumatic fever	Delayed sequela of pharyngitis
Post-streptococcal glomerulonephritis	Immunologically-mediated sequela of pharyngitis or skin infections

Table adapted from [3,125].
